# Can respiratory muscle training therapy effectively manage obstructive sleep apnea syndrome after stroke? A protocol of systematic review and meta-analysis

**DOI:** 10.1097/MD.0000000000020589

**Published:** 2020-06-12

**Authors:** Shu-wen Guo, Chang-fei Dai, Liang Yu, Xiong-fei Zhao

**Affiliations:** a Department of Neurology-Neuroelectrophysiology, Cardio-Crebrovascular Disease Hospital of Meishan, Meishan, Sichuan; b Department of Neurology, Xianyang Hospital of Yan’an University, Xianyang; c Department of Neurology, Sichuan Provincial People's Hospital, Chengdu, China.

**Keywords:** effectiveness, obstructive sleep apnea syndrome, respiratory muscle training, safety

## Abstract

**Background:**

This study will explore the effectiveness and safety of respiratory muscle training therapy (RMTT) for the treatment of patients with obstructive sleep apnea syndrome (OSAS) after stroke.

**Methods:**

In this study, we will systematically and comprehensively search Cochrane Library, PubMed, EMBASE, WANGFANG, VIP, Chinese Biomedical Literature Database, and China National Knowledge Infrastructure for relevant literature from their inception to March 1, 2020 without any limitations to language and publication status. We will consider any randomized controlled trials focusing on the effectiveness and safety of RMTT for the treatment of patients with OSAS after stroke. The study quality will be checked using Cochrane risk of bias tool, and statistical analysis will be performed utilizing RevMan 5.3 software.

**Results:**

This study will summarize and synthesize the current evidence of RMTT for the treatment of patients with OSAS following stroke.

**Conclusion:**

The findings of this study will assess the present evidence for the benefits and harms of RMTT for treating OSAS after stroke, and will inform clinical practice and future research.

**PROSPERO registration number:**

PROSPERO CRD42020170355.

## 1 Introduction

Stroke is one of the most common neurological diseases.^[1–3]^ Patients with such condition often experience a variety of complications, including brain edema, pneumonia, urinary tract infection, seizures, bedsores, pain, deep venous thrombosis, depression, anxiety, and obstructive sleep apnea syndrome (OSAS), especially for OSAS.^[4–18]^ It has been estimated that about 57% of stroke patients suffer from OSAS.^[19,20]^ Multiple modalities have been used to treat this condition, such as respiratory muscle training therapy (RMTT), as measured by parameters of polysomnography or any related outcome indicators.^[21–32]^ However, there has been no attempt to systematically summarize clinical evidence supporting the use of RMTT in treating patients with OSAS after stroke. Thus, it is important to establish evidence of the utilization of the RMTT modality in the management of stroke patients with OSAS. The goal of this study is to assess the benefits and harms of RMTT for treating OSAS after stroke.

## 2 Methods

### 2.1 Study registration

This protocol has been registered on PROSPERO (CRD42020170355). We report this study according to the guidelines of the Preferred Reporting Items for Systematic Reviews and Meta-Analysis Protocol statement.^[33,34]^

### 2.2 Criteria for considering studies

#### 2.2.1 Type of studies

Randomized controlled trials (RCTs) irrespective of language and publication status which assessing the effectiveness and safety of RMTT for OSAS after stroke will be included in this study. However, we will exclude any other studies, except the RCTs.

#### 2.2.2 Type of participants

Any patients who were diagnosed as OSAS after stroke will be included in this study, regardless their ethnic background, sex, and age.

#### 2.2.3 Type of interventions

Any forms of RMTT for OSAS after stroke has been assigned to the participants in the experimental group.

Any control intervention, except RMTT has been used to subjects in the comparator group.

#### 2.2.4 Type of outcomes

The primary outcome is severity of sleep apnea, as measured using Apnea-Hypopnea Index, or other relevant scales.

The secondary outcomes include parameters of polysomnography; quality of life, as assessed by Pittsburgh Sleep Quality Index, or other associated tools; and any expected or unexpected adverse events.

### 2.3 Search strategy

We will search Cochrane Library, PubMed, EMBASE, WANGFANG, VIP, Chinese Biomedical Literature Database, and China National Knowledge Infrastructure systematically and comprehensively from their inception to March 1, 2020 regardless language and publication status. The following search terms will be used: stroke, cerebrovascular accident, ischemic stroke, hemorrhagic stroke, post stroke, after stroke, sleep apnea syndromes, sleep apnea, obstructive, sleep disordered breathing, sleep disorder, breath stops, respiratory, muscle, training, education, and exercise. We have built an example of search strategy for Cochrane Library database in Table 1. Similar detailed search strategies will be adapted to other databases.

**Table 1:**
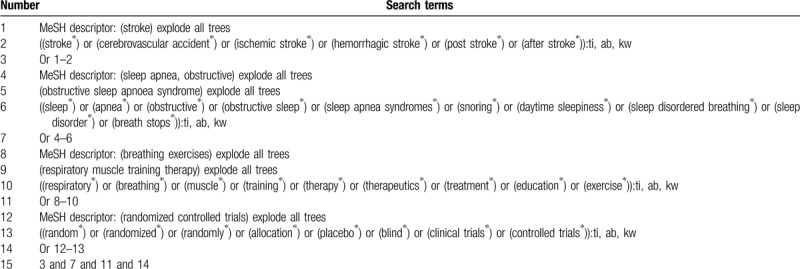
Search strategy for Cochrane Library database.

In addition, we will also search conference abstracts, dissertations, ongoing studies from clinical trial registry website, and reference lists of relevant studies.

### 2.4 Data selection and extraction

#### 2.4.1 Study selection

Two researchers will independently scan titles and abstracts of all retrieved records, and all irrelevant studies will be removed. Then, we will search all relevant full-paper study reports/publications. We will solve any divergences between 2 researchers through discussion; and a third researcher will be consulted to make decision if required. We will note all removed studies with specific reasons. The whole process of study selection will be shown in a flowchart.

#### 2.4.2 Data extraction

Two independent researchers will extract data from included trials, respectively. Any disagreements between 2 researchers will be solved by discussion with a third researcher. We will extract the following data: first author, time of publication, country, patient characteristics, diagnostic criteria, inclusion and exclusion criteria, sample size, study setting, study methods, intervention and comparator details, outcome measurements, adverse events, and funding information. We will contact original corresponding authors to inquire any unclear or missing data by email.

### 2.5 Study quality assessment

We will assess the risk of bias for each eligible study using Cochrane Risk of Bias Tool (Cochrane Community, Chichester, UK). We will evaluate each included study through 7 aspects, and each one is classified as low, unclear, or high risk of bias. Two researchers will independently check the risk of bias for each study, respectively. Any differences between 2 researchers will be solved by a third researcher.

### 2.6 Data analysis

In this study, RevMan 5.3 software (Cochrane Community, London, UK) will be applied for statistical analysis. We will express continuous values as mean difference or standardized mean difference and 95% confidence intervals (CIs), and dichotomous values as risk ratio and 95% CIs. *I*^2^ statistic will be used to investigate the statistical heterogeneity across included studies. Value of *I*^2^ ≤ 50% means homogeneity, while value of *I*^2^ > 50% indicates obvious heterogeneity. We will use a fixed-effects model if *I*^2^ ≤ 50, and will apply a random-effects model if *I*^2^ > 50%. Where homogeneity is sufficient between groups across included studies, we will perform a meta-analysis. Otherwise, we will conduct subgroup analysis to explore any possible reasons for the obvious heterogeneity. Additionally, we will report outcome results as a narrative summary in accordance with the Guidance on the Conduct of Narrative Synthesis in Systematic Reviews.

### 2.7 Subgroup analysis

We will carry out subgroup analysis based on the different study methodological quality, interventions, comparators, and outcomes.

### 2.8 Sensitivity analysis

We will conduct sensitivity analysis to identify the robustness of outcome results by excluding high risk of bias studies.

### 2.9 Reporting bias

When sufficient studies are included, we will check reporting bias using Funnel plot^[35]^ and Egger regression test.^[36]^

### 2.10 Ethics and dissemination

Ethical approval is not needed since no original data will be used in this study. The results of this study are expected to be published at a peer-reviewed journal.

## 3 Discussion

Despite several studies focus on exploring the benefits and harms of RMTT for treating OSAS after stroke, no single systematic review has investigated its effect and safety. This study will provide a comprehensive summary of the direct evidence on the effectiveness and safety of RMTT for the treatment of patients with OSAS after stroke. We expect this study that will improve our understanding of the limitations of the current available study to inform the treatment of RMTT for OSAS after stroke. In addition, its results may help clinical practice, patients, and researchers for design of future clinical trials.

## Author contributions

**Conceptualization:** Shu-wen Guo, Chang-fei Dai, Liang Yu, Xiong-fei Zhao.

**Data curation:** Shu-wen Guo, Chang-fei Dai, Xiong-fei Zhao.

**Formal analysis:** Shu-wen Guo, Chang-fei Dai, Liang Yu, Xiong-fei Zhao.

**Investigation:** Chang-fei Dai.

**Methodology:** Shu-wen Guo, Xiong-fei Zhao.

**Project administration:** Chang-fei Dai.

**Resources:** Shu-wen Guo, Liang Yu, Xiong-fei Zhao.

**Software:** Shu-wen Guo, Liang Yu, Xiong-fei Zhao.

**Supervision:** Chang-fei Dai.

**Validation:** Shu-wen Guo, Chang-fei Dai, Liang Yu, Xiong-fei Zhao.

**Visualization:** Shu-wen Guo, Chang-fei Dai, Xiong-fei Zhao.

**Writing – original draft:** Shu-wen Guo, Chang-fei Dai, Liang Yu, Xiong-fei Zhao.

**Writing – review & editing:** Shu-wen Guo, Chang-fei Dai, Liang Yu, Xiong-fei Zhao.
